# Lower content of cerebrospinal fluid chemokines in patients with cancer cachexia

**DOI:** 10.1590/acb414726

**Published:** 2026-08-03

**Authors:** Marcos Fraga Fortes, Gabriela Noronha Fortes, Marcos Noronha Fortes, Fernanda Christoffel Gomes, Marilia Seelaendar, Ariene Murari Soares de Pinho, Joyce de Cassia Rosa de Jesus, José Pinhata Otoch

**Affiliations:** 1 Universidade de São Paulo – Faculdade de Medicina – Departamento de Cirurgia – São Paulo (SP) – Brazil.; 2 Universidade de Santo Amaro – Faculdade de Medicina – Santo Amaro (SP) – Brazil.

**Keywords:** Neoplasms, Cachexia, Cerebrospinal Fluid, Chemokines, Vascular Endothelial Growth Factor A, Inflammation

## Abstract

**Purpose:**

Cancer-associated cachexia (CC) is characterized by involuntary weight loss and poor prognosis. Central nervous system (CNS) inflammation has been linked to CC symptoms. This study evaluated inflammatory mediators in the cerebrospinal fluid (CSF) of patients with CC.

**Methods:**

Volunteers were recruited in Santarém, PA, Brazil, and divided into three groups: patients undergoing herniorrhaphy or cholecystectomy (n = 43)—control; cancer without cachexia (CWC, n = 13); and CC (n = 49). Blood and CSF were collected before anesthetic injection.

**Results:**

CSF vascular endothelial growth factor (VEGF) and monocyte chemoattractant protein-1 (MCP-1) levels were reduced in CC (*p* < 0.0001; *p* 0.00017) and CWC (*p* = 0.0269; *p* = 0.0421) compared to control. CSF IP-10 was lower in CC (*p* = 0.0048). In CWC, CSF MCP-1 correlated with serum C reactive protein (rs = 0.78, *p* = 0.0098), leukocytes (rs = 0.72, *p* = 0.0235), lymphocytes (rs = -0.67, *p* = 0.0390) and monocytes (rs = -0.82, *p* = 0.0058). In CC, CSF interleukin (IL)-8 correlated with leukocytes (rs = 0.48, *p* = 0.0032) and MCP-1 with neutrophils (rs = 0.36, *p* = 0.0265) and eosinophils (rs = -0.37, *p* = 0.0218). CC patients showed lower quality of life than CWC (functional scale *p* = 0.0087; symptoms scale *p* = 0.0386).

**Conclusion:**

Cancer was related to changes in CSF cytokine levels, indicating that both tumor presence and cachexia modulate central inflammatory signaling. Correlations between CSF chemokines and blood immune cells suggest systemic interaction between peripheral immunity and the CNS.

## Introduction

Cancer-associated cachexia is a multifactorial syndrome that leads to weight loss and contributes to a lower quality of life and mortality^1^. Patients with cancer cachexia experience an ongoing anorexia with a depletion of energy stores^2^. The administration of an agonist of ghrelin, a hormone secreted by the stomach, to patients with cancer showed an increase in food intake and body weight^3^. Animal studies have shown that ghrelin agonist modulates hypothalamic neurons that control appetite. These findings show that central nervous system (CNS) control of food intake has great potential to mitigate the effects of this syndrome. However, studies regarding the control of appetite in humans with cancer cachexia are lacking.

Increased inflammatory mediators in the brain are correlated symptoms of cachexia, as total food intake and muscle mass4. In animal models of cachexia, pro-inflammatory cytokines, such as interleukin (IL)-1β and tumor necrosis factor (TNF)-α, can induce a hypothalamic response that leads to anorexia and catabolism of peripheral tissues^1^. CNS control of appetite seems to be influenced by inflammatory proteins that can activate receptors in the pro-opiomelanocortin and Agouti-related protein neurons1. Moreover, IL-1β can activate the hypothalamus-pituitary-adrenal axis with consequently increase in muscle proteolysis^1^. Experimental studies have demonstrated that cancer cachexia is associated with central nervous system inflammation, including myeloid cell infiltration, activation of inflammatory signaling pathways, and alterations in feeding behavior and energy balance^4-6^.

Chemokines are a class of small proteins that are mainly known as chemotactic mediators in the immune system but also have several other homeostatic functions, which include the pro-inflammatory activation of peripheral immune cells^7-9^. In the CNS, chemokines have shown abilities related to neuromodulation, neuroendocrine regulation and acts as neurotransmitters and may be released by neurons, astrocytes, or microglia^9,10^.

Chemokine content in the cerebrospinal fluid (CSF) has been evaluated in several psychiatric conditions, as depression and Alzheimer’s disease^11,12^. Although its growing interest regarding its role in the CNS, chemokines are still not intensely studied in the context of cancer cachexia and the quality of life of patients with this syndrome^13^. We have previously reported that patients with cancer cachexia presented changes in the content of CSF chemokines^13^. Here, we aimed to evaluate the content of inflammatory mediators in the CSF of patients with cancer cachexia. Moreover, we deepened the analysis of these results and correlated these CSF chemokines with other relevant parameters associated this syndrome.

## Methods

### Patients

The study was approved by the Ethics Committee of the Medical School of the Universidade de São Paulo (CAAE 78485517.4.3001.5467). Samples were collected from 105 patients of both sexes, aged between 18 and 85 years old. Participants were divided into three groups:

- Control: subjects without cancer or cachexia who required herniorrhaphy or cholecystectomy (n = 43);
- CWC: patients with cancer without cachexia (n = 13);
- CC: patients with cancer and cachexia (n = 49).

The Suppl. Mat. 1 (https://doi.org/10.5281/zenodo.20835596) shows the types of surgery that the participants of the control group were submitted to. The types of cancer from the CWC and CC are presented in Suppl. Mat. 2 (https://doi.org/10.5281/zenodo.20835596).

The volunteers were recruited in the Hospital Regional do Baixo Amazonas Dr. Waldemar Penna (HRBA), Santarém, PA, Brazil. The study protocol had no interference with the therapeutic medical conduct. The patients were informed about the study, invited to participate, and included when the informed consent form was signed. At the time of surgery, during spinal anesthesia, CSF was collected from all patients preceding the injection of anesthetic.

The inclusion criteria were: age between 18 and 85; cancer patients with or without cachexia treated at HRBA; patients undergoing curative or palliative surgery with the possibility of collecting CSF during anesthesia; patients that agreed to participate and signed the informed consent form. For the control group, the inclusion criteria were: patients undergoing elective herniorrhaphy or cholecystectomy; between 18 and 85 years old; with no history of current weight loss; and no associated inflammatory condition.

The exclusion criteria were: the presence of sepsis (defined according to the criteria of the Society of Critical Care Medicine and American College of Chest Physicians^14^); the presence of severe coagulopathy with international normalized ratio > 2 or thrombocytopenia < 40,000; decompensated heart failure; primary hematologic diseases; moderate renal failure (creatinine above 2 mg/dL); dependence on circulatory or ventilatory support; pregnancy; chronic use of anti-inflammatory drugs; participation in another study; use of chemotherapy in the last three months; puncture accident with compromise of the CSF sample; use of anticoagulant medication; increased CSF pressure due to any etiology; infectious disease or previous CNS surgery.

### General parameters

Complete blood count, albumin, and C reactive protein (CRP) data were obtained from the medical records. All these analyses were performed in the hospital itself, prior to the surgery. The European Organization for Research and Treatment of Cancer-Quality of Life Questionnaire Core 30 (QLQ-C30) was performed on patients with cancer^15^.

### Criteria for diagnosing cachexia

Cachexia was identified based on the criteria proposed by Evans et al.^16^. To its identification, changes were considered in body weight, as involuntary weight loss in the last six months greater than or equal to 5% of the initial body mass; body mass index (BMI) less than 20 kg/m^2^ for patients under 65 years old and less than 22 kg/m^2^ for patients aged 65 older, and at least three of the following criteria: decreased muscle strength; the presence of fatigue; anorexia; low fat-free body mass index; serum albumin less than 35 g/dL; serum CRP concentration higher than 5mg/L; and anemia (Hb < 12 g/dL).

### Cerebrospinal fluid collection

Two mL of CSF were collected and aspirated by subarachnoid puncture during the anesthetic procedure, before the infusion of any drug through this route. The routine preparation for spinal anesthesia included fasting for 12 hours before the procedure, and an empty bladder immediately before anesthesia. In the surgical center, the patient received intravenous sedation. Then, lying in lateral decubitus and bent forward, a fine needle was inserted into his back, after anesthetizing the skin and subcutaneous tissue with lidocaine, in the area between the last lumbar vertebrae (L3/L4; L4/L5), up to the spinal canal. After CSF collection, the anesthetic was injected within the same puncture. The samples were stored in liquid nitrogen and in a -80°C freezer.

### Quantification of inflammatory proteins and growth factors in cerebrospinal fluid

Inflammatory cytokines were quantified in the CSF with the Multiplex system using the Human Cytokine/Chemokine Magnetic Bead Panel Cat. HCYTMAG-60 K-PX30 (Merck-Millipore) is designed to analyze: interferon (IFN)-α2, IFN-γ, IL-1α, IL-1β, IL-1ra, IL-2, IL-3, IL-4, IL-5, IL-6, IL-7, IL-8, IL-10, IL-12p40, IL-12p70, IL-13, IL-15, IL-17A, interferon gamma-induced protein-10 (IP-10, also known as C-X-C motif chemokine ligand 10), TNF-α, TNF-β, granulocyte-colony-stimulating factor (G-CSF), granulocyte-macrophage colony-stimulating factor (GM-CSF), vascular endothelial growth factor (VEGF), monocyte chemoattractant protein-1 [MCP-1, also known as chemokine (C-C motif) ligand 2—CCL2], macrophage inflammatory protein (MIP)-1α (also known as chemokine (C-C motif) ligands 3—CCL3), MIP-1β (also known as chemokine (C-C motif) ligands 4—CCL4), C-C motif chemokine 11 (CCL11). Only IL-8, IP-10, VEGF, MCP-1, and MIP-1β were satisfactorily quantified in the CSF.

### Statistical analysis

A preliminary analysis was performed to verify that the normality assumptions were not violated (Shapiro-Wilk’s test). Kruskal-Wallis’ tests followed by Dunn’s test were used to compare non-parametrical. Unpaired T-test was used to compare parametrical data (QLQ-C30 functional and symptoms scales). Significance was considered at *p* < 0.05. The results obtained were expressed as median (first quartile; third quartile) when non-parametrical and mean and standard deviation when data presented parametrical distribution and analyzed with GraphPad Prism Software (version 8.4.3 Copyright^©^, 1992–2020).

## Results

The CC group was composed of 49 individuals, with 22 female participants; the CWC group presented 13 participants, with seven women; and the control group comprised 43 individuals, with 23 females. No difference was observed for the sex distribution in the three groups. The CC group was older and had a lower BMI than the control group, as shown in Table 1. CC and CWC presented lower hemoglobin compared to the control. CC showed more inflammation with higher CRP, neutrophils, and lymphocytes than the control and CWC groups. Moreover, CC had lower albumin than the control and CWC groups, and higher platelets compared to the control.

**Table 1:** General parameters of the three groups<tfn href="tfn01">*</tfn>.

	Control	CWC	CC	*p* -value
Number	43	13	49	
Age (years old)	44 (38–61)	60 (41.5–67.5)	59 (48–71.5)	0.0027^a^
Sex (Female/male)	23/20	7/6	22/27	0.6747χ
Body weight (kg)	69.5 (63.75–74.25)	60 (58.5–75.5)	53 (47–62)	< 0.0001^a^
BMI (kg/m^2^)	24.86 (23.44–25.99)	22.94 (21.59–24.68)	20.57 (17.82–23.12)	< 0.0001^a^
CRP (mg/dL)	2.2 (0.6–4.9)	4 (1.2–6.3)	42 (22.85–83.10)	< 0.0001^a^,^b^
Albumin (g/dL)	4 (3.8–4.3)	3.9 (3.55–4.05)	3.1 (2.6–3.6)	< 0.0001^a^; 0.0019^b^
Hemoglobin (g/dL)	14.60 (13.3–15.4)	12.7 (10.9–13.8)	9.8 (8.9–11.6)	0.0246^c^; < 0.0001^a^
Leukocytes (mm^3^)	7,700 (5,900–10,199)	7,300 (5,500–8,550)	9,200 (6,400–11,300)	0.0389**
Basophils (%)	0.3 (0.2–0.5)	0.3 (0.2–0.5)	0.3 (0.2–0.4)	0.4679
Eosinophils (%)	2.7 (1.5–5.5)	2.6 (1.7–5.15)	1.9 (0.85–4.4)	0.1168
Neutrophils (%)	56.8 (50.7–63.8)	54.4 (47.9–70.05)	71.5 (63.45–85.15)	< 0.0001^a^; 0.0120^b^
Lymphocytes (%)	33.6 (23.5–40.4)	28.8 (21.85–39.4)	17.7 (7.6–25.3)	< 0.0001^a^; 0.0144^b^
Monocytes (%)	6.1 (4.9–7.33)	6.0 (4.75–7.45)	6.3 (5.2–7.2)	0.9601
Platelets (x109/L)	265 (209–292)	269 (198–350)	327 (216–474)	0.0029^a^
QLC-C30 functional scale	-	73.06 ± 21.19	50.40 ± 18.85	0.0087
QLQ-C30 symptoms scale	-	30.45 ± 24.50	48.72 ± 18.83	0.0386

* Comparisons were performed using the Kruskal-Wallis’ test followed by Dunn’s *post-hoc* test;

a control *versus* cancer cachexia group;

b cancer without cachexia group *versus* cancer cachexia group;

c control versus cancer without cachexia group;

χ χ^2^ test;

** no differences in the Dunn’s *post-hoc* test;

BMI: body mass index; CRP: C reactive protein; QLQ-C30: European Organization for Research and Treatment of Cancer-Quality of Life Questionnaire Core 30.

Source: Elaborated by the authors.

**Figure 1:**
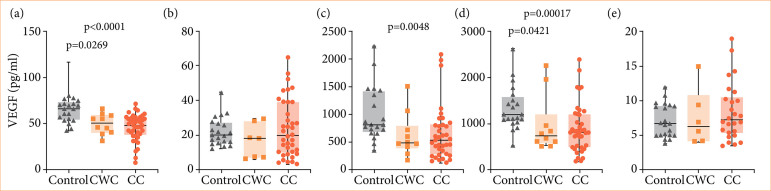
VEGF and chemokines in cerebrospinal fluid of the control, CWC, and CC groups. (a) VEGF; (b) IL-8; (c) IP-10; (d) MCP-1; (e) MIP-1β. Comparisons were performed using the Kruskal-Wallis’ test followed by Dunn’s post-hoc test.

VEGF: vascular endothelial growth factor; CWC: cancer without cachexia group; CC: cancer cachexia group; IL-8: interleukin 8; IP-10: IFN-γ-inducible protein 10; MCP-1: monocyte chemoattractant protein-1; MIP-1: macrophage inflammatory protein-1 beta. Source: Elaborated by the authors.

**Figure 2:**
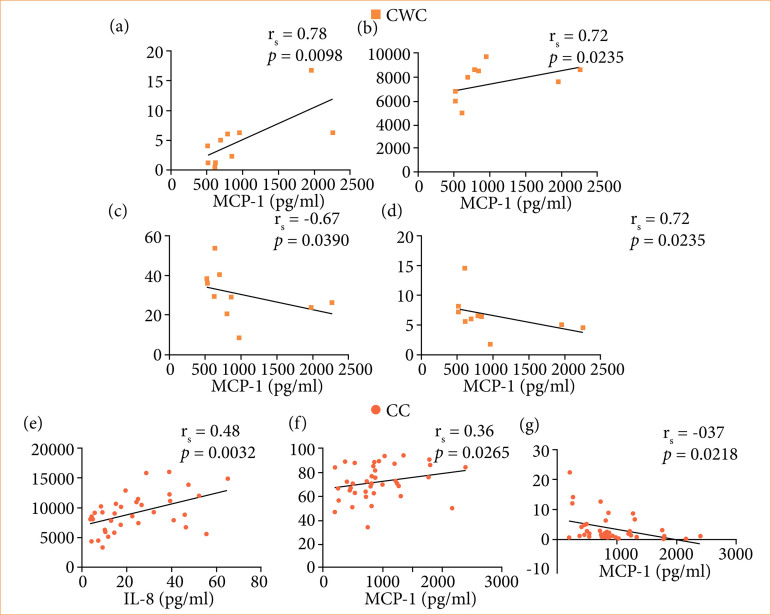
Correlations between chemokines in cerebrospinal fluid and serum CRP and blood immune cells in CWC and CC groups. (a) CRP and MCP-1 in CWC; (b) leucocytes and MCP-1 in CWC; (c) lymphocytes and MCP-1 in CWC; (d) monocytes and MCP-1 in CWC; (e) leucocytes and IL-8 in CC; neutrophils and MCP-1 in CC; eosinophils and MCP-1 in CC. Comparisons were performed using the Spearman’s correlation test.

CWC: cancer without cachexia group; CC: cancer cachexia group; CRP: C reactive protein; MCP-1: monocyte chemoattractant protein-1; IL-8: interleukin 8. Source: Elaborated by the authors.

The CSF of patients with cancer—CWC and CC groups—showed lower content of VEGF and MCP-1 compared to the control group, as indicated in Fig. 1. CSF IP-10 was lower in the CC group compared to the control group. The correlation analysis in the CWC group demonstrated that CSF MCP-1 was positively associate with serum CRP and total leucocytes and negatively correlated with the percentage of lymphocytes and monocytes, as shown in Fig. 2.

Regarding the CC group, circulating leucocytes and neutrophils were positively associated with CSF IL-8 and MCP-1, respectively, and eosinophils were negatively associated with CSF MCP-1. No significant correlations were found for the control group, as seen in Suppl. Mat. 3 (https://doi.org/10.5281/zenodo.20835596).

**Figure 3:**
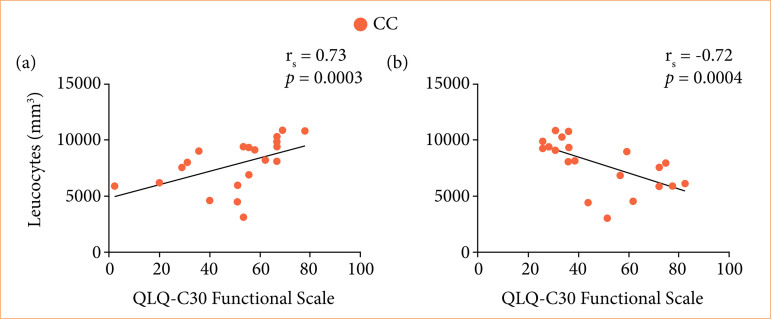
Association of EORT QLQ-C30 scales with leucocytes in the CC group. (a) Leucocytes and QLQ-C30 functional scale. (b) Leucocytes and QLQ-C30 symptoms scale. Comparisons were performed using the Spearman’s correlation test.

CC: cancer cachexia group; QLQ-C30: European Organization for Research and Treatment of Cancer-Quality of Life Questionnaire Core 30. Source: Elaborated by the authors.

Associations between EORT QLQ-C30 scales and the other variables were also analyzed. Only the CC group presented significant correlations. QLQ-C30 functional scale was positively associated with leucocytes, and QLQ-C30 symptoms scale was negatively associated with leucocytes. These results indicate that CC patients with higher functionality presented more circulating leucocytes and CC patients with high levels of symptomatology presented lower blood leucocytes, as shown in Fig. 3.

## Discussion

Patients with CC presented lower content of CSF VEGF, IP-10, and MCP-1 compared to the control group, and patients with CWC showed lower amounts of CSF VEGF and MCP-1 compared to the control group. These results indicate that a tumor’s presence altered the signaling protein content in the CSF. We have previously reported similar results with lower CSF VEGF and MCP-1 content in CC and CWC groups compared to control patients^13^. Furthermore, in our study, patients with cachexia showed a lower quality of life with a higher presence of symptoms measured by the QLQ-C30.

CSF chemokines were associated with blood immune cells in patients with cancer with and without cachexia, but differently in each group. Patients with CC had a positive association of CSF IL-8 with leukocytes and CSF MCP-1 with neutrophils and a negative association of CSF MCP-1 and eosinophils. Furthermore, only patients with CC showed an association between blood immune cells and the EORT QLQ questionnaire, with blood leukocytes being positively associated with the functional scale and negatively associated with the symptoms scale. Therefore, in this study, higher blood leukocytes were associated with higher quality of life in patients with cancer cachexia. Patients with cancer without cachexia presented a positive association of CSF MCP-1 with serum CRP and blood leukocytes and a negative association of CSF MCP-1 with lymphocytes and monocytes.

MCP-1 can induce migration and proliferation of microglia without directly inducing a pro-inflammatory state^17^. When injected, MCP-1 can induce mechanical allodynia showing its role in nociception^18^. Patients with malignant glioma presented higher CSF MCP-1 compared to patients with benign glioma and control subjects^19^. Children with acute lymphoblastic leukemia showed an increase in CSF IL-6 and MCP-1 after chemotherapy induction treatment and increased CSF IL-6, MCP-1, and TNF-α during the consolidation phase compared to the levels measured at the diagnosis^20^. Moreover, MCP-1 has been implicated in CNS inflammation after injury of axons, ischemia, and dorsal root ganglion chronic compression^8^.

CSF IP-10 was lower in patients with Alzheimer’s disease and in patients with subjective cognitive decline compared to patients with mild cognitive impairment^21^. IP-10 is involved in the pathogenesis of inflammatory demyelinating neuropathies, as showed by its increased expression in the peripheral nervous system of patients with Gillain-Barré syndrome and patients with chronic inflammatory demyelinating polyradiculoneuropathy^22^. Patients with cancer cachexia have shown higher plasma levels of IP-10 compared to patients with cancer without cachexia^23^. In a mouse model of pancreatic ductal adenocarcinoma (PDAC), IP-10 gene expression was downregulated in the area postrema and upregulated in the hippocampus, and this same work showed that culture of microglia exposed to PDAC conditioned media did not increase IP-10 gene expression^4^. However, the role of CSP IP-10 or even CNS IP-10 in patients with cancer cachexia has not been fully explored so far.

In the CNS, chemokines are important mediators of the homeostatic crosstalk between brain cells^24^. It has been reported an increase in the CNS chemokines and cytokines after lipopolysaccharides (LPS) injection together with a decrease in food intake in mice^6^. Post-mortem analysis of brains from patients with cancer cachexia showed increased microglia density in the hypothalamus and a higher phagocytic activity in the caudate nucleus microglia compared to patients with cancer without cachexia^25^. Therefore, there is evidence of increased CNS inflammation related to cancer cachexia. The data presented here showed that CSF MCP-1 and IP-10 were lower in the CC group compared to control individuals. Blood leucocytes and neutrophils were positively associated with IL-8 and MCP-1, respectively, in patients with cancer cachexia. The lower CSF chemokine content in patients with cachexia is a new finding only reported by another study of our research group with a subset of the same cluster of patients^13^. These new finds indicate that CSF chemokines may be sequestrated to peripheral tissues due to the presence of a tumor or that chronic high-grade inflammation may lead to a compensatory downregulation of chemokine production in the CNS.

CSF chemokines—eotaxin-1, MIP-1β, MCP-1, MCP-4, and thymus and activation-regulated chemokine—have been previously reported to be low in patients with psychiatric disorders in conjunction with a suicide attempt compared to control subjects^11^. In this study, authors found a correlation between low CSF chemokine and pain^11^. Lower CSF chemokine content seems associated with cognitive alterations, which may impact the disease perception and quality of life. Regarding VEGF, higher CSF levels of this growth factor are associated with a less executive function decline over time, less hippocampal atrophy, and more optimal brain aging in individuals with normal cognition, mild cognitive impairment, and Alzheimer’s disease^26^.

## Conclusion

Patients with cancer cachexia presented lower CSF VEGF, MCP-1 and IP-10 compared to control subjects, and patients with CWC had lower CSF VEGF and MCP-1 compared to the control group. These results suggest that the presence of tumor was able to interfere with the content of inflammatory mediators in the CSF. The potential mechanisms leading to lower CSF chemokines in cancer patients need further research. CSF chemokines in both groups were correlated with blood immune cells, suggesting a systemic interaction between peripheral immune cells and the CNS.

## Data Availability

The data that support the findings of this study are available within the article and from the corresponding author upon reasonable request.
